# In this month’s Bulletin 

**DOI:** 10.2471/BLT.22.000422

**Published:** 2022-04-01

In editorials this month, Viroj Tangcharoensathien et al. (238) call for papers for a special issue on the impact of climate change on biodiversity, agriculture and health. Lisa L Carter et al. (239) propose the next decade’s global genomic surveillance strategy for pathogens with pandemic and epidemic potential. Tedros Adhanom Ghebreyesus et al. (240) present WHO recommendations for resilient health systems.

Tatum Anderson (243–244) reports on the environmental implications of medical waste generated by the COVID-19 response. Benjie Foscablo talks to Gary Humphreys (245–246) about the pressures of nursing during the COVID-19 pandemic, why nurses are leaving the profession, and the urgent need to improve working conditions.

## Burkina Faso 

### Preventing chronic Hepatitis B 

Alice Nanelin Guingané et al. (256–267) evaluate a screening programme for partners and children of pregnant women.

## India

### Diagnosis of measles and rubella 

Deepa Sharma et al. (247–255) describe laboratory network expansion.

### Creating an evidence base for environmental health

Tanwi Trushna & Rajnarayan R Tiwari (281–285) introduce a new research institute.

## Global

### Curtailing tobacco, alcohol and unhealthy foods 

Anne Marie Thow et al. (268–275) examine safeguards in trade and investment agreements.

### Towards better policy-making

Susan P Sparkes et al. (276–280) show how political economy analysis can improve health programmes. 

### Water, sanitation and hygiene

Sophie Budge et al. (286–288) track the evolution of environmental sanitation.

### “Too much, too late” 

Siem Zethof et al. (289–291) use data on stillbirths to interpret caesarean section rates.

